# Fine-Tuning Large Language Models for Motivational Interviewing in Health Behavior Change: Development and Evaluation Study

**DOI:** 10.2196/89077

**Published:** 2026-06-24

**Authors:** Runze Hu, Yang Yang, Yihang Yang, Jingqi Kong, Jiahui Luo, Wenyu Yang, Jing Chen, Jingyao Liu, Huiqun Zeng, Lei Zhang, Zheng Liu

**Affiliations:** 1 Department of Maternal and Child Health, School of Public Health, Peking University, No. 38 Xueyuan Road, Haidian District, Beijing, 100191, China, 86 010-82801222; 2 Peking University China Center for Health Development Studies, School of Public Health, Peking University, Beijing, China; 3 Taomi AI4Health Lab, Beijing, China

**Keywords:** motivational interviewing, large language models, health education, behavior change, fine-tuning

## Abstract

**Background:**

Motivational interviewing (MI) is an effective counseling approach for promoting health behavior change, but its scalability is constrained by the need for highly trained human counselors. Large language models (LLMs) may provide a scalable way to support MI counseling, but evidence remains limited, especially for Chinese MI resources and evaluations based on standardized MI fidelity frameworks.

**Objective:**

This study aimed to develop Chinese large language models for motivational interviewing (MI-LLMs) and evaluate whether MI-focused fine-tuning could improve their ability to generate counseling responses consistent with MI principles.

**Methods:**

We first curated 5 publicly available Chinese psychological counseling datasets and assessed sampled conversations in terms of comprehensiveness, professionalism, authenticity, and safety. The 2 highest-scoring datasets, CPsyCounD and PsyDTCorpus, were selected for MI-style data construction. Using GPT-4 with a structured MI-informed prompt, we transformed 2040 multiturn counseling conversations into MI-style dialogs. Among these, 2000 dialogs were used for training and 40 for testing. Three Chinese-capable open-source LLMs (Baichuan2-7B-Chat, ChatGLM-4-9B-Chat, and Llama-3-8B-Chinese-Chat-v2) were fine-tuned with low-rank adaptation on the training dataset and were referred to as MI-LLMs. Automatic evaluation was conducted on the testing dataset using Bilingual Evaluation Understudy–4 (BLEU-4) and Recall-Oriented Understudy for Gisting Evaluation (ROUGE) metrics. Manual evaluation was conducted using the Motivational Interviewing Treatment Integrity Coding Manual 4.2.1. Thirty simulated counseling dialogs generated by the MI-LLMs were compared with 30 real MI dialogs sampled from AnnoMI and translated into Chinese. Two trained graduate student raters coded global scores and behavior counts, from which summary scores were subsequently calculated.

**Results:**

In automatic evaluation, fine-tuning substantially improved BLEU-4 and ROUGE scores across all 3 models compared with the base models. In manual evaluation, the MI-LLMs achieved technical and relational global scores, as well as total MI-adherent ratios that approached those of real MI dialogs. The MI-LLM based on ChatGLM-4-9B-Chat showed the strongest overall global performance. However, MI-LLMs produced fewer complex reflections and had lower reflection-to-question ratios than real MI dialogs.

**Conclusions:**

This study provides preliminary evidence that MI focused fine-tuning can help Chinese LLMs acquire core counseling behaviors consistent with MI principles. It also offers a scalable approach for constructing MI style dialog resources in Chinese. Nevertheless, current MI-LLMs should be regarded as early-stage tools for supporting, rather than replacing human counselors. Future work should expand real MI training data and strengthen the complex reflective skills of MI-LLMs. Further studies are needed to evaluate their effectiveness, acceptability, and safety in health behavior change settings in the real world.

## Introduction

Chronic diseases, such as cardiovascular disease, diabetes, and obesity, have become significant global health challenges [1,2]. Health behavior promotion is fundamental to the prevention and management of most of these chronic diseases. However, achieving long-term maintenance of behavior change through such interventions remains challenging [3-8]. One line of research highlights the importance of enhancing individuals’ intrinsic motivation [9].

To this end, motivational interviewing (MI), an evidence-based counseling approach to facilitating behavior change [10], has been proven effective in promoting health behavior change across a variety of behaviors [11,12], such as smoking cessation [13], weight loss [14], substance use [15], physical activity [16,17], and chronic disease self-management [18]. However, MI’s effectiveness depends strongly on the professional therapist’s experiences and skills, which require extensive training and continuous practice over several years [19,20]. These demanding training requirements not only constrain the scalability of MI but also make it difficult for practitioners to maintain Motivational Interviewing Treatment Integrity (MITI) over time [21].

The rise of large language models (LLMs), such as GPT-4, has introduced a promising tool for addressing these challenges. LLMs have demonstrated advanced natural language processing capabilities and have been successfully applied in a variety of health-related tasks [22,23]. However, existing research on the integration of MI with artificial intelligence or conversational agents faces several key limitations, including small, self-constructed datasets [24], rule-based system architectures [25-28], and outcome evaluation that is not adherent to standardized MI fidelity frameworks [26,28,29].

In light of these limitations, this study aimed to (1) provide a scalable pathway for constructing a Chinese MI-style dialog dataset, (2) train fine-tuned LLMs to master the core skills of MI, and (3) assess the MI abilities of fine-tuned LLMs using both automatic and manual evaluation based on the MITI Coding Manual 4.2.1 [30].

## Methods

### Study Design

**Figure 1.**
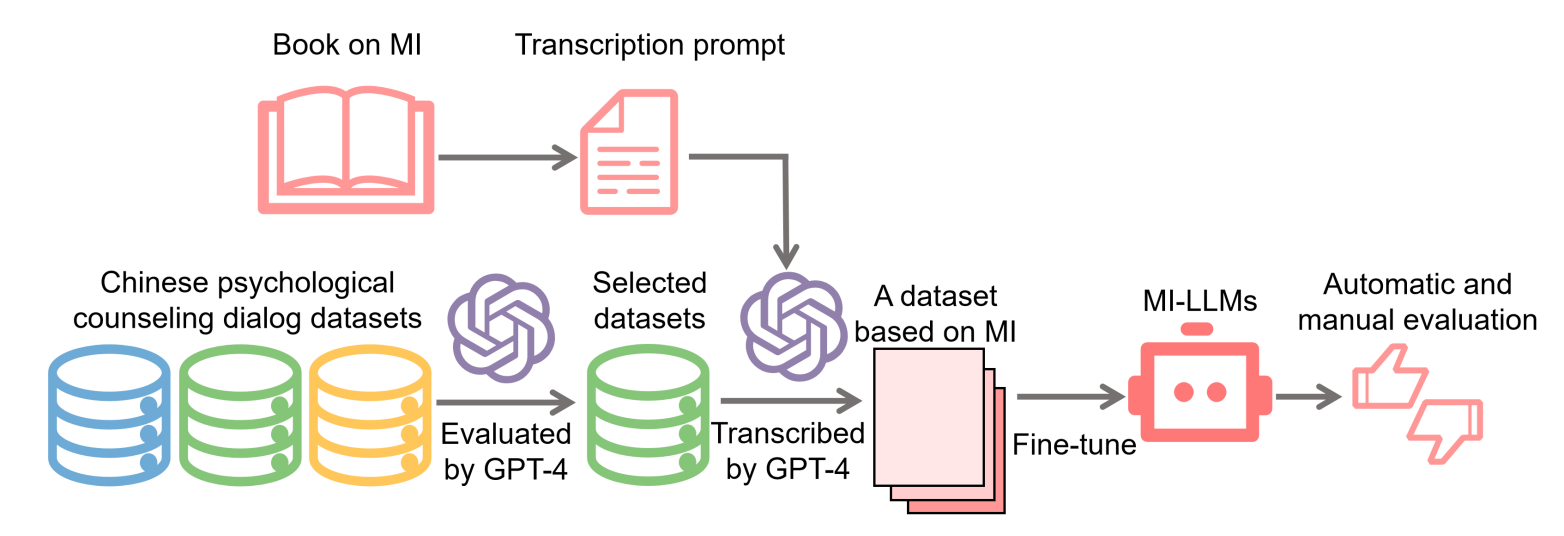
Overall design of this study. MI: motivational interviewing; MI-LLM: large language model for motivational interviewing.

Figure 1 shows the overall design of this study. We first collected available Chinese psychological counseling dialog datasets through online searches and evaluated randomly sampled conversations to select high-quality sources. Since these datasets did not contain structured MI content, we designed an MI-informed prompt to transform ordinary psychological counseling dialogs into MI-style multiturn dialogs. Using this dataset, we fine-tuned the LLM and developed the large language model for motivational interviewing (MI-LLM) model. Finally, through both automatic and manual evaluations, we comprehensively assessed whether the MI-LLMs initially had the potential to promote healthy behaviors by conducting MI.

### Screening and Evaluating the Counseling Dialog Dataset

We searched Chinese psychological counseling dialog datasets from platforms including Kaggle, GitHub, Hugging Face, and OpenDataLab. Afterward, we randomly selected 50 conversations from each dataset and referenced CPsyCounD’s automated evaluation methodology to evaluate the quality of these conversations [31]. Specifically, this evaluation criterion included 4 aspects: comprehensiveness, professionalism, authenticity, and safety. Details of these 4 aspects of the evaluation criteria are shown in Table S1 of Multimedia Appendix 1. In the interest of data diversity, we decided to proceed with the top 2 datasets based on their aggregate scores.

### Transcription of the Counseling Dialogs

According to an authoritative book in the MI field [10], we summarized the 4 basic tasks and interview techniques of MI to prepare a transcription prompt for GPT-4, a closed-source proprietary large language model developed by OpenAI, to transcribe Chinese psychological counseling conversations into MI-style dialogs. The transcription prompt template was strictly developed based on prompt engineering principles [32], ensuring both structural rigor and functional appropriateness. Specifically, it was structured around the following key components: role, task objective, the 4 core tasks of MI, key techniques of MI, transformation steps, guiding principles, and an output format example. The specific prompt was shown in Multimedia Appendix 1. After transcription, all generated dialogs were manually screened to remove replies that were irrelevant, incoherent, unsafe, or clearly inconsistent with MI principles. These transcriptions based on GPT-4 were conducted in Beijing, China, between January 1, 2025, and February 28, 2025.

### Original Model Information

Three open-source LLMs with strong Chinese language capabilities—Baichuan2-7B-Chat, ChatGLM-4-9B-Chat, and Llama-3-8B-Chinese-Chat-v2—were downloaded from Hugging Face for fine-tuning and testing.

Baichuan2-7B-Chat [33] is an open-source LLM launched by Baichuan Intelligence in December 2023, which was trained with a high-quality corpus of 2.6 trillion tokens. ChatGLM-4-9B-Chat [34] is an open-source version of the pretrained model in the GLM-4 series, launched by Smart Spectrum AI in June 2024, and has demonstrated superior performance beyond Meta-Llama-3-8B in multifaceted dataset measurements. Meta-Llama-3-8B [35] is an open-source LLM released by Meta in April 2024, using a high-quality corpus of 15 trillion tokens for pretraining with excellent performance. However, since only 5% of the training corpus of Meta-Llama-3-8B is non-English, it often answers Chinese prompts in English during inference. Instead of using the original model of Llama3, we use Llama-3-8B-Chinese-Chat-v2 [36], a model based on Meta-Llama-3-8B, which provides a further 100,000 tokens of Chinese corpus for preference training, so that the model’s Chinese language ability has been greatly developed.

To evaluate the models’ language comprehension and reasoning capabilities, we compiled the models’ scores on the following 3 key benchmarks. First, massive multitask language understanding (MMLU) is a large-scale multitask benchmark mainly used for evaluating English language models across 57 diverse tasks, measuring broad language understanding and reasoning abilities. Second, Chinese MMLU (CMMLU) is a multitask benchmark for evaluating comprehensive Chinese language understanding, including commonsense reasoning, mathematical reasoning, and sentiment analysis. Third, Chinese evaluation (C-Eval) is a benchmark designed to assess Chinese language understanding and generation capabilities. It covers multiple tasks such as reading comprehension, reasoning, and translation.

All benchmarks consist solely of multiple-choice questions. Model performance is reported as accuracy; therefore, higher scores indicate better model capability. For better comparison, we also collected the publicly reported scores of GPT-4 and Meta-Llama-3-8B on these benchmarks.

The specific scores for each model are shown in Table 1. The results showed that ChatGLM-4-9B-Chat performs strongly on C-Eval and CMMLU, demonstrating superior capability in Chinese-language applications. Although Llama-3-8B-Chinese-Chat-v2 and Baichuan2-7B-Chat achieved relatively lower scores, they still exhibited sufficient Chinese comprehension and reasoning abilities to serve as base models for fine-tuning in this study.

**Table 1.** Models’ scores on 3 key benchmarks and the corresponding testing methods.

Model	MMLU^a^	CMMLU^b^	C-Eval^c^	Evaluation setting	Source
Baichuan2-7B-Chat^d^	52.9	55.0	54.4	5-shot	[33]
ChatGLM-4-9B-Chat^d^	72.4	75.1	75.6	Not mentioned	[34]
Llama-3-8B-Chinese-v2^d^	63.7	52.4	49.8	5-shot	[37]
GPT-4^e^	83.9	70.3	68.4	5-shot^f^	[33]
Meta-Llama-3-8B^e^	65	50.8	49.4	5-shot	[37]

a MMLU: massive multitask language understanding.

b CMMLU: Chinese multitask language understanding.

c C-Eval: Chinese evaluation.

d Models fine-tuned in this study.

e Models included for comparison.

f 5-shot means that when the model performs a task, it is provided with 5 examples as references or learning guides. These examples help the model recognize patterns or rules in the task, thereby improving its reasoning and predictions.

### Methods of Fine-Tuning

Our study used the integrated tool LLaMA-Factory [38], which was specifically designed for LLM—a unified framework for efficient fine-tuning, evaluation, and deployment of LLMs. In addition, low-rank adaptation (LoRA) was adopted to fine-tune the LLMs. LoRA improves training efficiency by adding trainable LoRA matrices to the original model while keeping most pretrained parameters frozen, thereby reducing memory consumption compared with full-parameter fine-tuning. During training, we set the following key hyperparameters: a learning rate of 1.0×10^–4^, which was gradually reduced using a cosine learning rate scheduler; a per-device training batch size of 1; and a gradient accumulation step size of 8 to achieve a larger effective batch size with limited memory. The number of training epochs was set to 3 to avoid overfitting; the warmup ratio was set to 0.1 to gradually increase the learning rate; and BF16 precision was used to accelerate training and reduce memory usage. During the training process, logs were recorded every 10 steps, the model was saved every 500 steps, and the change curve of the loss function was plotted.

Through fine-tuning the 3 models (Baichuan2-7B-Chat, ChatGLM-4-9B-Chat, and Llama-3-8B-Chinese-Chat-v2), we obtained corresponding domain-specific models. Based on the initials of MI and LLM, we named them MI-LLMs.

### Automatic and Manual Evaluation

#### Overview

After fine-tuning, we evaluated the MI-LLMs using automatic generation metrics and manual MITI–based coding. The inference and evaluation runs for the base models and fine-tuned MI-LLMs were conducted on the High-Performance Computing Platform of Peking University in Beijing, China, between February 1, 2025, and March 31, 2025.

#### Automatic Evaluation

For automatic evaluation, we constructed round-based test samples to compare each model-generated counselor response with the corresponding reference counselor response. This design allowed us to evaluate model performance at each client-counselor exchange while preserving the prior dialog history as context.

Our test dataset consisted of complete, transcribed MI-style psychological counseling dialogs, with each dialog corresponding to 1 full client-counselor session. We refer to these full dialogs as test dialogs. When constructing the round-based test samples, we split each transcribed MI-style dialog into rounds, taking each client volley as the model input and using all preceding rounds as dialog history to provide context. One round was defined as a single client-counselor exchange. For every round-level test sample, we attached a fixed instruction prompt: “You are a psychological counselor with 20 years of experience. Your aim is to help clients solve psychological problems through professional Motivational Interviewing counseling.”

Formally, for a test dialog with *i* rounds (ie, *i* client-counselor pairs), we denote it as {(*q_k_,r_k_*) | k = 1,2,...,*i*}, where *q_k_* and *r_k_* are the client’s volley and the counselor’s reply at round *k*, respectively. At round *k*, the dialog history is defined as:


(1) $$h_{k}=\{(q_{j},r_{j})|j=1,2,\ldots ,k-1\}$$

where *h_k_* represents all previous client queries and counselor responses. Conditioning on the fixed MI prompt *P*_MI_, the model generates a response *rˆ_k_* according to:


(2) $${\hat{r}}_{k}=\left\{ \begin{array}{ll} f_{LLM}\left(P_{MI},q_{k}\right), & k=1,\\ f_{LLM}\left(P_{MI},h_{k},q_{k}\right), & 1§lt;k\leq i, \end{array}\right.$$

where *f*_LLM_(·) denotes the inference process of the language model. Thus, each test dialog in the test dataset gives rise to *i* round-level test samples in an Alpaca-style triplet form (instruction, input, and output, including the history and the most recent from the client).

For example, in a 3-round smoking-cessation conversation, round 1 uses the client’s first volley (“Hello, I feel that I have been under a lot of pressure recently, and I can’t help smoking”) as the input, together with the fixed MI prompt, and the corresponding counselor reply as the target output. In round 2, the input consists of the client’s second volley (“Yes, I really want to quit smoking... but I just can’t quit it”), the same fixed prompt, and the full dialog history from round 1 (the client’s first volley and the counselor’s response), with the counselor’s second reply as the target. In round 3, the client’s third volley, expressing worry about withdrawal reactions, is combined with the same fixed prompt and the cumulative history from rounds 1 and 2, and the counselor’s third reply serves as the target output. A detailed example of this round-based division is shown in Table S2 of Multimedia Appendix 1.

We first fed the round-based test samples constructed above into each model to obtain its generated responses, *rˆ_k_* for every turn *k*. For automatic evaluation, we then compared each generated response *r̂_k_*with its corresponding reference counselor response *r̂_k_*. The outputs of the original base models and the MI-LLMs were treated as generated texts, while the counselor’s volleys in the test dataset served as reference texts. Using LLaMA-Factory, we computed Bilingual Evaluation Understudy–4 (BLEU-4), Recall-Oriented Understudy for Gisting Evaluation–1 (ROUGE-1), ROUGE-2, and ROUGE-L between each generated response and its corresponding reference. These automatic metrics were used to quantify the improvement in the models’ ability to produce MI-consistent counseling responses that more closely match real counselors’ language and better handle multiturn psychological dialogs. Detailed definitions and implementation settings of the automatic metrics are provided in Multimedia Appendix 1.

#### Manual Evaluation

In the manual evaluation, 2 researchers role-played standardized clients. Specifically, we predefined for each standardized client the target behavior to be changed, the specific manifestations of that behavior, the underlying reasons for the behavior (with the provision that only by addressing these reasons would the motivation for change be triggered), and the client’s baseline readiness to change. The 2 researchers were each randomly assigned several standardized client profiles and then conducted the dialogs with the LLMs according to their assigned profiles. For each MI-LLM, we obtained 10 independent and complete multiround conversations, resulting in a total of 30 simulated counseling dialogs across the 3 MI-LLMs.

In addition, we used real MI dialogs from the AnnoMI dataset as a reference set [39]. AnnoMI is a publicly available collection of expert-annotated MI dialogs, originally derived from audiorecorded counseling sessions and presented in the form of textual transcripts. We randomly sampled 30 dialogs from AnnoMI, which were originally in English, and translated them into Chinese to ensure language consistency with the conversations generated by MI-LLMs and to facilitate uniform manual evaluation.

Before manual evaluation, all dialogs were deidentified, formatted into the same transcript structure, and randomly ordered. The raters were not informed whether each dialog came from a real MI session or an MI-LLM–generated interaction. In total, 60 written multiround transcripts, including 30 simulated counseling dialogs and 30 real MI dialogs, were manually evaluated. We also categorized and counted the topics of 2 sets of dialogs.

Manual coding was conducted according to the MITI Coding Manual 4.2.1 [30]. The evaluation indicators included (1) global scores on four dimensions of cultivating change talk, softening sustain talk, partnership, and empathy; and (2) behavior counts, including giving information, persuading with permission, asking questions, simple reflections, complex reflections, affirming, seeking collaboration, emphasizing autonomy, persuading, and confronting. After completing the assessment of these indicators, we used the following equation to calculate summary scores for quantitative evaluation. The assignment of points for each indicator is shown in Table 2. All MITI coding was carried out by 2 graduate students: 1 in psychology and 1 in medicine. To ensure coding accuracy, both raters first independently downloaded and studied the MITI Coding Manual 4.2.1. They then completed 2 online training sessions led by a trainer certified by the Motivational Interviewing Network of Trainers. The first session reviewed the MITI manual with illustrative examples. In the second session, the raters coded 4 standardized sample dialogs (141 volleys in total). For global scores of 4 dialogs, the Pearson correlations between each rater and the reference standard scores were 0.64 and 0.65, respectively, while the interrater correlation was 0.84. For Cohen κ of behavior coding across the 141 volleys, the κ values between each rater and the standard codes exceeded 0.58, and the interrater κ reached 0.69. Based on these results, the trainer confirmed that the raters were adequately prepared to proceed with formal coding.

**Table 2.** Method for calculating summary scores in manual evaluation.

Indicator	Calculating method
Complex reflection ratio	=Complex reflection/(Simple reflections+Complex reflection)
Reflection-to-question ratio (R:Q)	=Total reflections/(Total questions)=(Simple reflections+Complex reflection)/(Total questions)
Technical global	=(Cultivating change talk+Softening sustain talk)/2
Relational global	=(Partnership+Empathy)/2
Total MI-adherent ratio^a^	=(Seeking collaboration+Affirming+Emphasizing autonomy)/(Seeking collaboration+Affirming+Emphasizing autonomy+Confronting+Persuading)

a Motivational Interviewing Treatment Integrity Coding Manual 4.2.1 used 2 indicators to evaluate the conformity and nonconformity of motivational interviewing (MI).

For the real MI dialogs and the dialogs generated by each MI-LLM, we separately calculated the mean and SD of the global scores, behavior counts, and summary scores. These descriptive statistics allowed us to directly compare the counseling performance of human MI practitioners with that of the 3 MI-LLMs:

Total MI-adherent=Seeking collaboration+Affirming+Emphasizing autonomy

Total MI-nonadherent=Confronting+Persuading

However, these 2 indicators are related to the length of the dialog. The longer the dialog, the larger the values of these 2 indicators might be. Therefore, we adopted a ratio to normalize and compare the conformity of MI across different dialog lengths:

Total MI-adherent ratio=Total MI-adherent/(Total MI-adherent+Total MI nonadherent)

### Reporting

We used the CHART (Chatbot Assessment Reporting Tool) checklist (Checklist 1) to ensure that our reporting was comprehensive [40].

### Ethical Considerations

Ethics board review was not sought for this study because it did not involve the recruitment of external human participants, clinical intervention, identifiable private information, or confidential patient records. The study used publicly available and deidentified dialogue datasets in accordance with their public availability and citation requirements. According to Article 32 of the Measures for Ethical Review of Life Science and Medical Research Involving Humans [41], research using legally obtained publicly available data or anonymized information may be exempt from ethics review when it does not cause harm to humans and does not involve sensitive personal information or commercial interests. In the manual evaluation, researchers role-played standardized clients using predefined hypothetical profiles solely for model testing. Therefore, informed consent from external participants was not applicable.

## Results

### Results of Screening Chinese Psychological Counseling Dialog Datasets

**Figure 2.**
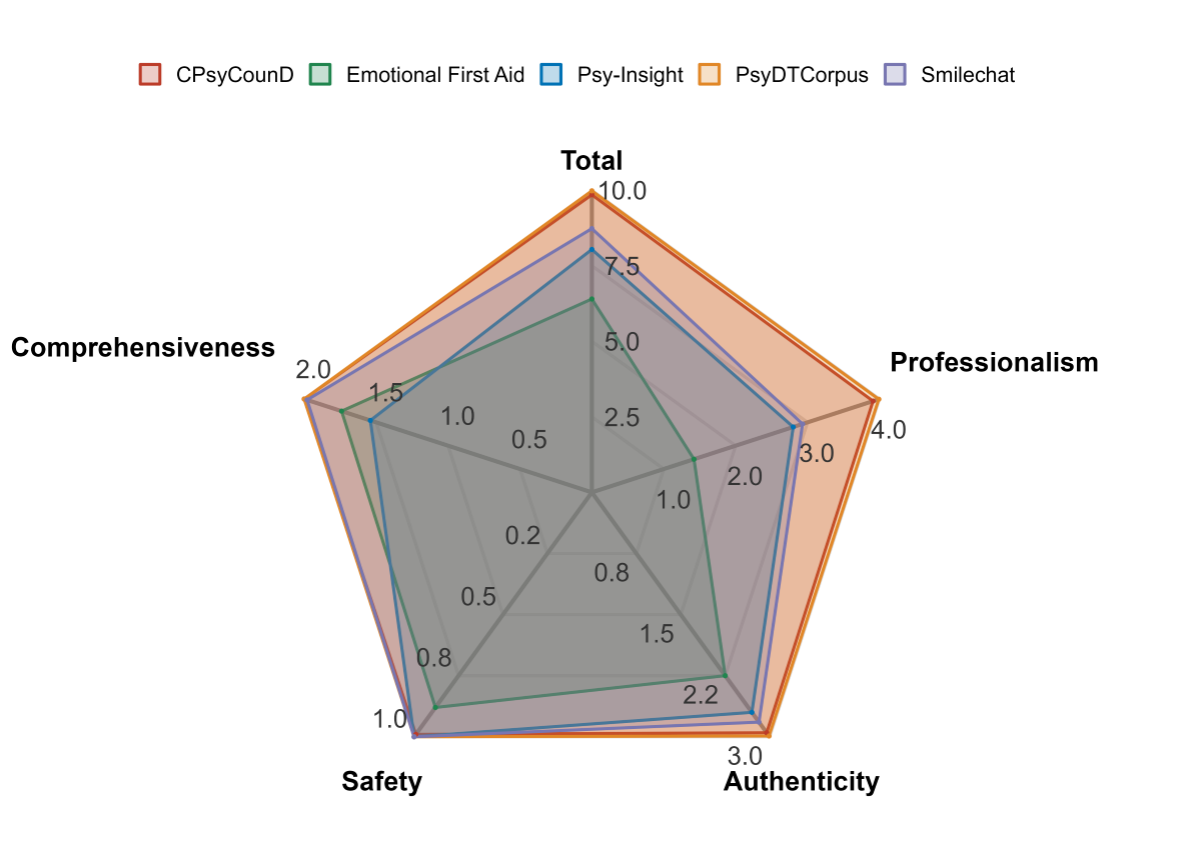
The evaluation results of GPT-4 on the scores of 4 perspectives and the total score across 5 Chinese psychological counseling dialog datasets.

We obtained 5 Chinese psychological counseling dialog datasets: CPsyCounD [31], Emotional First Aid Dataset [42], Psy-Insight [43], PsyDTCorpus [44], and Smilechat [45]. The quality of these datasets was evaluated from 4 perspectives: comprehensiveness, professionalism, authenticity, and safety. The results are shown in Figure 2. Among the 5 datasets, CPsyCounD and PsyDTCorpus achieved 2 highest overall scores. Therefore, we constructed the training and test datasets based on these 2 datasets.

### Construction of MI-Style Datasets

First, we extracted 1520 multiround dialogs from CPsyCounD and 520 dialogs from PsyDTCorpus, based on their optimal quality compared with the other 3 datasets. Second, we transcribed them into MI-style dialogs by using prompt engineering with GPT-4, obtaining 2040 transcribed multiturn dialogs in total. Third, from 2040 transcribed multiturn dialogs, we randomly selected 2000 of them as the training dataset and the remaining 40 as the test dataset.

Specifically, for CPsyCounD and PsyDTCorpus, the training dataset and the test dataset, we calculated the average number of dialog rounds, the average number of words in each client volley, and the average number of words in each counselor volley. The results are shown in Table 3.

**Table 3.** Statistical analysis of dialog features in the CPsyCounD, PsyDTCorpus, and the motivational interviewing–style training dataset.

Datasets	Dialog rounds, mean (SD)	Words per counselor volley, mean (SD)	Words per client volley, mean (SD)
CPsyCounD	7.65 (2.07)	29.95 (18.22)	47.76 (20.78)
PsyDTCorpus	18.08 (3.15)	28.67 (11.41)	53.74 (17.96)
Training dataset	7.97 (4.56)	29.08 (16.38)	57.33 (17.20)
Test dataset	7.53 (4.12)	29.21 (18.62)	56.42 (15.90)

### Results of Automatic Evaluation

**Figure 3.**
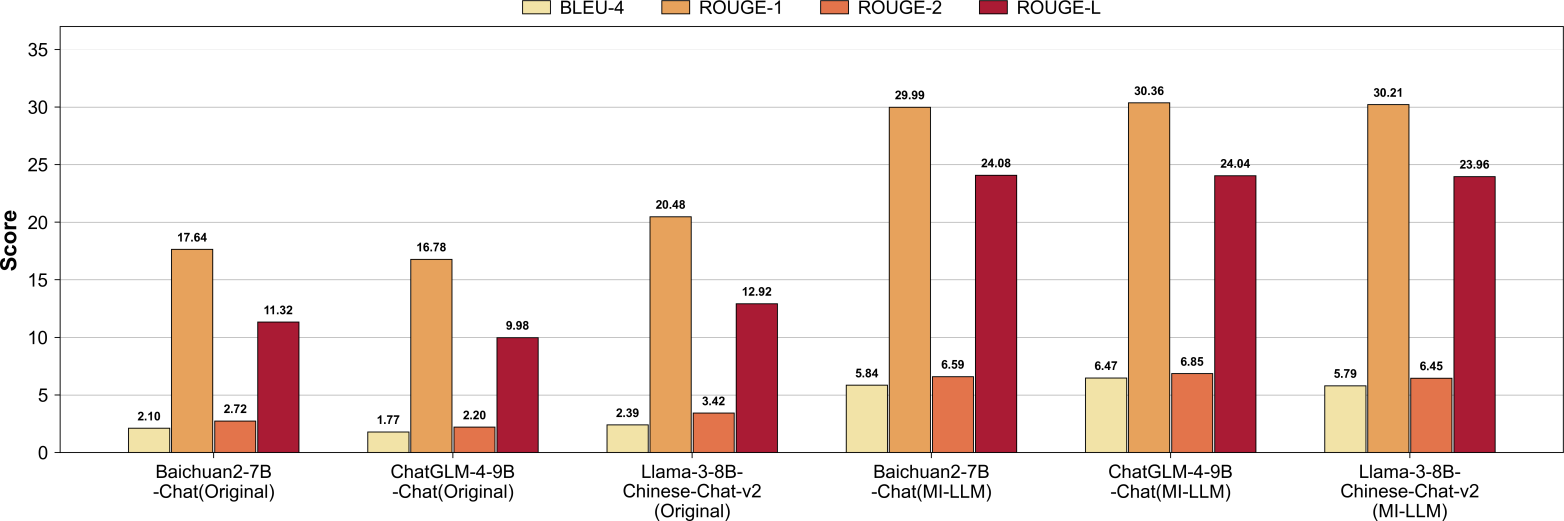
The automatic evaluation results of large language models for motivational interviewing (MI-LLMs) and the original model on the test datasets.

We divided the 40 test dialogs into 367 round-based samples to evaluate the fine-tuning effect of the model. As shown in Figure 3, all fine-tuned MI-LLMs performed better than their corresponding base models across BLEU-4 and ROUGE metrics. Specifically, the BLEU-4 score of the Baichuan2-7B-Chat–based MI-LLM increased from 2.10 for the original Baichuan2-7B-Chat to 5.84; for the ChatGLM-4-9B-Chat–based MI-LLM, it increased from 1.77 to 6.47; and for the Llama-3-8B-Chinese-Chat-v2–based MI-LLM, it increased from 2.39 to 5.79. Regarding the ROUGE series metrics (ROUGE-1, ROUGE-2, and ROUGE-L), the performance of the MI-LLMs was likewise consistently better than that of the original models.

### Results of Manual Evaluation

Because the actual temporal duration was not directly comparable between transcript-based real MI dialogs and text-based LLM-generated dialogs, we compared dialog length using the average number of dialog rounds and the average volley length of clients and counselors. As shown in Table 4, the real MI dialogs contained more dialog rounds than the simulated counseling dialogs generated by the 3 MI-LLMs. Differences were also observed in volley length. Real MI dialogs showed shorter counselor volleys but longer client volleys on average compared to the simulated dialogs.

**Table 4.** Statistical analysis of dialog features in real MI^a^ dialogs and simulated counseling dialogs.

Source	Dialogs, n	Dialog rounds, mean (SD)	Words per counselor volley, mean (SD)	Words per client volley, mean (SD)
Real MI dialogs	30	57.93 (65.95)	24.26 (13.04)	21.38 (9.74)
Simulated counseling dialogs from 3 MI-LLMs^b^				
Baichuan2-7B-Chat–based	10	18.30 (7.27)	49.11 (6.57)	14.80 (5.67)
ChatGLM-4-9B-Chat–based	10	17.40 (5.17)	52.28 (10.30)	11.45 (4.93)
Llama-3-8B-Chinese-Chat-v2–based	10	18.90 (5.80)	43.79 (8.70)	10.71 (5.80)

a MI: motivational interviewing.

b MI-LLM: large language model for motivational interviewing.

These findings suggest differences in turn-taking and volley structure between real MI and MI-LLM-generated dialogs. However, because the real MI dialogs were translated speech-derived transcripts, whereas the MI-LLM dialogs were originally generated as Chinese text–based interactions, the differences in Table 4 may also reflect language, translation, topic, dataset source, and communication-modality effects rather than human-LLM differences alone.

We categorized the topics of 30 real MI dialogs and 30 simulated counseling dialogs from 3 MI-LLMs. Both the real MI dialogs and the simulated counseling dialogs generated by the MI-LLMs primarily focused on behavior change. In particular, topics such as weight loss or diet management, increasing exercise, and reducing dependence on external objects or substances (eg, mobile phones, alcohol, or other addictive behaviors) were represented in both sets of dialogs. Details are shown in Table 5.

Based on the MITI framework, the manual evaluation results for simulated counseling dialogs generated by MI-LLMs and for real MI dialogs—including the mean and SD for each indicator—are summarized in Table 6.

**Table 5.** Topics of real MI^a^ dialogs and simulated counseling dialogs from MI-LLMs^b^.

Topics	Counts
Real MI dialogs	
Increasing exercise	6
Chronic disease self-management	6
Weight loss or diet management	3
Reducing recidivism	3
Reducing drug use	2
Reducing alcohol use	2
Quitting smoking	1
Other	7
Simulated counseling dialogs from 3 MI-LLMs	
Weight loss or diet management	4×3
Reducing mobile phone use	2×3
Improving insomnia	2×3
Increasing exercise	1×3
Reducing excessive consumption	1×3

a MI: motivational interviewing.

b MI-LLM: large language model for motivational interviewing.

**Table 6.** The manual evaluation results of global scores, behavior counts, and summary scores.

Evaluation indicators	Simulated counseling dialogs from MI-LLMs^a^			Real MI^b^ dialogs
	Baichuan2-7B-Chat–based	ChatGLM-4-9B-Chat–based	Llama-3-8B-Chinese-Chat-v2–based	
Global scores, mean (SD)				
Cultivating change talk	4.00 (0.00)	4.00 (0.00)	4.00 (0.00)	3.97 (0.89)
Softening sustain talk	3.89 (0.33.)	4.00 (0.00)	3.90 (0.32)	3.87 (0.86)
Empathy	3.78 (0.44)	4.00 (0.00)	3.80 (0.67)	4.07 (1.11)
Partnership	3.44 (0.88)	4.00 (0.00)	4.00 (0.67)	3.83 (0.95)
Behavior counts, mean (SD)				
Giving information	3.22 (1.56)	4.00 (2.75)	4.50 (1.78)	1.07 (1.48)
Persuading with permission	0.33 (0.50)	0.00 (0.00-0.00)	0.10 (0.32)	0.80 (1.56)
Asking questions	15.56 (7.24)	14.20 (5.15)	16.00 (5.10)	13.43 (11.68)
Simple reflections	13.50 (6.08)	13.25 (4.58)	13.10 (3.77)	14.17 (23.76)
Complex reflections	6.00 (2.52)	4.25 (2.03)	5.00 (2.33)	6.13 (8.54)
Total reflections	19.5 (8.50)	17.5 (6.09)	18.1 (5.74)	20.3 (28.15)
Affirming	4.44 (1.81)	3.75 (0.79)	3.35 (1.20)	3.30 (3.84)
Seeking collaboration	1.78 (0.57)	1.60 (0.52)	1.40(0.70)	1.53 (1.89)
Emphasizing autonomy	1.06 (0.39)	1.25 (0.26)	0.45 (0.50)	0.60 (0.86)
Persuading	3.83 (2.78)	2.30 (1.58)	3.40 (1.26)	1.57 (1.65)
Confronting	0.11 (0.22)	0.00 (0.00)	0.00 (0.00)	0.10 (0.40)
Summary scores, mean (SD)				
Complex reflections ratio	0.31 0.03)	0.24 (0.08)	0.26 (0.06)	0.37 (0.35)
Reflection-to-question ratio	1.27 (0.09)	1.25 (0.10)	1.11 (0.14)	1.44 (0.93)
Technical global	3.94 (0.17)	4.00 (0.00)	3.95 (0.16)	3.92 (0.80)
Relational global	3.61 (0.65)	4.00 (0.00)	3.90 (0.46)	3.95 (0.95)
Total MI-adherent ratio	0.75 (0.08)	0.78 (0.12)	0.72 (0.06)	0.73 (0.33)

a MI-LLM: large language model for motivational interviewing.

b MI: motivational interviewing.

In terms of global scores, the simulated dialogs all received consistently high global ratings on cultivating change talk, softening sustain talk, empathy, and partnership, with mean scores mostly around or above 3.5 and limited variability. Among them, the MI-LLM based on ChatGLM-4-9B-Chat achieved the strongest overall global performance. It reached a mean of 4.00 on all 4 global items and obtained Technical Global and Relational Global scores of 4.00, slightly surpassing the other 2 MI-LLMs and the real MI dialogs. Real MI dialogs achieved empathy scores that were marginally higher than those of the MI-LLMs, indicating that experienced human counselors still hold an advantage in conveying empathic understanding. For cultivating change talk and partnership, however, the average scores of human counselors were similar to those of MI-LLMs, suggesting that the models can already approximate human-level performance on key MI-consistent global dimensions.

In terms of behavior counts, asking questions and simple reflections were the 2 most frequent behaviors in both MI-LLM-generated and real MI dialogs. Complex reflections occurred most often in real MI dialogs, with Baichuan2-7B-Chat–based and Llama-3-8B-Chinese-Chat-v2–based MI-LLMs producing somewhat more complex reflections than the ChatGLM-4-9B-Chat–based model, but still slightly fewer than human counselors on average.

Compared with real MI dialogs, MI-LLMs produced substantially more instances of giving information and slightly more affirming behaviors, indicating a strong tendency to support clients through explanations and positive feedback. Certain MI-adherent behaviors, such as seeking collaboration and emphasizing autonomy, appeared at least as frequently in MI-LLM simulations as in real dialogs, whereas persuading with permission was more common in real MI dialogs. MI nonadherent behaviors, including persuading and confronting, were infrequent in both groups, and confronting was almost absent.

The dispersion of behavior counts also differed between groups: dialogs generated by MI-LLMs showed relatively stable behavior frequencies across conversations, as reflected by smaller SD values for several behavior count indicators, whereas real MI dialogs exhibited greater variability between dialogs, reflecting the flexible adaptation of strategies in real clinical practice.

In terms of summary scores, real MI dialogs showed a clear advantage in complex reflective skills. The complex reflections ratio was highest in real MI dialogs, exceeding the ratios observed in all 3 MI-LLMs, which suggests that human counselors are more likely to use reflections that add inference, meaning, or emotional elaboration rather than merely repeating content. Similarly, the reflection-to-question (R:Q) ratio was higher in real MI dialogs than in any of the MI-LLM-generated dialogs, indicating that human practitioners rely relatively more on reflective listening than on asking questions.

In contrast, MI-LLMs performed very well on MI adherence. The total MI-adherent ratio was highest for the ChatGLM-4-9B-Chat–based MI-LLM, followed by the Baichuan2-7B-Chat–based model, both at or above the level observed in real MI dialogs, while the Llama-3-8B-Chinese-Chat-v2–based model was slightly lower but still close. Taken together, these results suggest that MI-LLMs are highly capable of following MI-consistent guidelines and avoiding nonadherent behaviors, whereas human counselors retain an advantage in more nuanced skills, particularly the use of complex reflections and maintaining a higher balance of reflections over questions.

## Discussion

### Principal Findings

This exploratory study tested the feasibility of developing MI-LLMs through dataset screening, MI-style data construction, model fine-tuning, and multilevel evaluation. We first transformed high-quality Chinese psychological counseling dialogs into MI-style multiturn conversations using an MI-informed GPT-4 prompt, providing a scalable approach for constructing MI training data in a resource-limited language context. Based on this dataset, 3 open-source Chinese-capable LLMs were fine-tuned and evaluated. Both automatic and manual evaluations suggested that MI-LLMs acquired several core MI-consistent techniques and principles. They achieved technical and relational global scores and MI-adherent ratios close to those of real MI dialogs, although human practitioners still showed advantages in complex reflections and R:Q balance.

### Interpretation of Findings

Our findings first suggest that MI-style data construction is a feasible strategy for addressing the shortage of high-quality MI dialog corpora, especially in non-English contexts. Similar to recent efforts such as Korean MI and IC-AnnoMI [24,46], this study shows that LLM-assisted data augmentation can be used to expand MI-related dialog resources when expert-annotated MI corpora are limited.

Second, the results support the value of task-specific fine-tuning for improving the MI-consistent behaviors of general-purpose LLMs. Across all 3 base models, fine-tuning improved automatic generation metrics, and the manual MITI–based evaluation showed that MI-LLMs achieved global scores and summary scores similar to those of real MI dialogs. These findings are consistent with recent evidence that fine-tuning can improve the quality of automated therapy delivered by LLMs [47]. However, the improvements were more evident for basic MI-consistent behaviors than for advanced MI skills. Compared with human counselors, MI-LLMs still showed lower complex reflection ratios and lower R:Q ratios, suggesting that fine-tuning may help models learn MI-adherent language patterns more easily than deeper reflective listening and session-level counseling strategies.

Third, our findings add to emerging evidence that LLMs can generate responses broadly aligned with MI principles and may be adapted for scalable health behavior support [48]. Prior studies have shown that LLM-generated MI responses can be contextually appropriate and that fully generative MI chatbots may be feasible in health behavior domains [49]. In line with this literature, our results suggest that MI-LLMs may serve as a promising supplement for health behavior change support. Nevertheless, their limitations in complex reflections and R:Q balance indicate that current MI-LLMs should not yet be viewed as replacements for trained human counselors, but rather as early-stage tools requiring further optimization, safety evaluation, and real-world validation.

### Strengths and Limitations

This study has several strengths. First, it addresses an important language and resource gap by developing and evaluating Chinese MI-LLMs in a field still dominated by English-language datasets and systems. Second, the study proposes a practical and scalable data construction strategy by transforming existing Chinese psychological counseling dialogs into MI-style multiturn conversations. Third, 3 open-source LLMs with Chinese language capability were fine-tuned and evaluated, which improves the robustness of the findings compared with a single-model design. Fourth, the evaluation combined automatic generation metrics with manual MITI–based coding, allowing us to assess not only text similarity but also clinically meaningful MI process indicators. Finally, the comparison with real MI dialogs from AnnoMI provided a more interpretable benchmark than model-to-model comparisons alone and helped identify specific skill gaps, especially in complex reflections and R:Q balance.

Several limitations should be noted. First, the training corpus was relatively small and was constructed through GPT-4-assisted transformation, which may limit the authenticity and generalizability of the generated dialogs. Second, the evaluation was based on researcher-simulated clients and written transcripts. Although dialogs were deidentified, standardized, randomly ordered, and rated without disclosing their source, complete blinding could not be guaranteed because raters might have inferred the source from observable dialog features. The comparison between translated English AnnoMI dialogs and originally Chinese MI-LLM dialogs may have been influenced by language, cultural, and communication-pattern differences. In addition, the use of written transcripts without audio or nonverbal cues, together with the small number of raters, means that the MITI-based results should be interpreted as preliminary process-based evidence rather than definitive evidence of clinical MI competence. Third, this study was not validated in real-world counseling or health behavior change settings, which limits the strength of evidence regarding the effectiveness, acceptability, and safety of MI-LLMs in actual use. Therefore, we could not assess how users might respond to MI delivered by nonhuman agents over time, including potential psychosocial risks. Although MI-LLMs may generate language that appears consistent with MI principles, text-based MI consistency should not be equated with human-delivered MI or a genuine counseling relationship, particularly because MI-LLMs lack access to tone of voice, facial expressions, body language, and other interpersonal cues that support face-to-face counseling. Future studies should evaluate potential unintended effects, including loneliness, emotional dependence, overreliance, reduced help-seeking from humans, and changes in real-world social support.

### Conclusions

In conclusion, this study provides preliminary evidence that MI-oriented fine-tuning can enable Chinese LLMs to produce counseling responses that approximate several core MI-consistent behaviors on MITI-based process measures. The findings suggest a scalable route for building MI dialog resources and developing AI-assisted tools for health behavior support. At the same time, current MI-LLMs remain limited in complex reflective listening, session-level strategy, real-world effectiveness, interpretability, and safety assurance. Future work should expand and diversify real MI training data, incorporate expert feedback or reinforcement learning from human preferences [50,51], evaluate models in rigorous real-world trials, including randomized controlled trials comparing MI-LLMs with human-delivered MI or usual care, and develop safeguards that make MI-LLMs more transparent, controllable, and clinically responsible.

## Supplementary material

10.2196/89077 Multimedia Appendix 1 Counseling datasets evaluation criteria, transcription prompts, and automatic evaluation details for large language models for motivational interviewing (MI-LLMs).

10.2196/89077 Checklist 1 CHART checklist.

## Abbreviations

BLEU-4: Bilingual Evaluation Understudy–4
LLM: large language model
LoRA: low-rank adaptation
MI: motivational interviewing
MI-LLM: large language model for motivational interviewing
MITI: Motivational Interviewing Treatment Integrity
MMLU: massive multitask language understanding
R:Q: reflection-to-question
ROUGE: Recall-Oriented Understudy for Gisting Evaluation

## References

[1] Budreviciute A, Damiati S, Sabir DK (2020). Management and prevention strategies for non-communicable diseases (NCDs) and their risk factors. Front Public Health.

[2] Noncommunicable diseases. World Health Organization.

[3] Fjeldsoe B, Neuhaus M, Winkler E, Eakin E (2011). Systematic review of maintenance of behavior change following physical activity and dietary interventions. Health Psychol.

[4] Kwasnicka D, Dombrowski SU, White M, Sniehotta F (2016). Theoretical explanations for maintenance of behaviour change: a systematic review of behaviour theories. Health Psychol Rev.

[5] Adab P, Pallan MJ, Lancashire ER (2018). Effectiveness of a childhood obesity prevention programme delivered through schools, targeting 6 and 7 year olds: cluster randomised controlled trial (WAVES study). BMJ.

[6] Ahern AL, Wheeler GM, Aveyard P (2017). Extended and standard duration weight-loss programme referrals for adults in primary care (WRAP): a randomised controlled trial. Lancet.

[7] Jay M (2025). Weight loss maintenance remains challenging-even with personalized support. JAMA Netw Open.

[8] Murray JM, Brennan SF, French DP, Patterson CC, Kee F, Hunter RF (2017). Effectiveness of physical activity interventions in achieving behaviour change maintenance in young and middle aged adults: a systematic review and meta-analysis. Soc Sci Med.

[9] Sheeran P, Wright CE, Avishai A, Villegas ME, Rothman AJ, Klein WMP (2021). Does increasing autonomous motivation or perceived competence lead to health behavior change? A meta-analysis. Health Psychol.

[10] Miller WR, Rollnick S (2023). Motivational Interviewing: Helping People Change and Grow.

[11] Frost H, Campbell P, Maxwell M (2018). Effectiveness of motivational interviewing on adult behaviour change in health and social care settings: a systematic review of reviews. PLoS ONE.

[12] Bischof G, Bischof A, Rumpf HJ (2021). Motivational interviewing: an evidence-based approach for use in medical practice. Dtsch Arztebl Int.

[13] Lindson-Hawley N, Thompson TP, Begh R (2015). Motivational interviewing for smoking cessation. Cochrane Database Syst Rev.

[14] Barnes RD, Ivezaj V (2015). A systematic review of motivational interviewing for weight loss among adults in primary care. Obes Rev.

[15] Schwenker R, Dietrich CE, Hirpa S (2023). Motivational interviewing for substance use reduction. Cochrane Database Syst Rev.

[16] Zhu S, Sinha D, Kirk M (2024). Effectiveness of behavioural interventions with motivational interviewing on physical activity outcomes in adults: systematic review and meta-analysis. BMJ.

[17] Harkin K, Apostolopoulos V, Tangalakis K, Irvine S, Tripodi N, Feehan J (2023). The impact of motivational interviewing on behavioural change and health outcomes in cancer patients and survivors. A systematic review and meta-analysis. Maturitas.

[18] Ekong G, Kavookjian J (2016). Motivational interviewing and outcomes in adults with type 2 diabetes: a systematic review. Patient Educ Couns.

[19] Miller WR, Moyers TB (2017). Motivational interviewing and the clinical science of Carl Rogers. J Consult Clin Psychol.

[20] Mitcheson L, Bhavsar K, McCambridge J (2009). Randomized trial of training and supervision in motivational interviewing with adolescent drug treatment practitioners. J Subst Abuse Treat.

[21] Bahri AA (2025). Motivational interviewing to promote healthy lifestyle behaviors: evidence, implementation, and digital applications. J Multidiscip Healthc.

[22] Guo Z, Lai A, Thygesen JH, Farrington J, Keen T, Li K (2024). Large language models for mental health applications: systematic review. JMIR Ment Health.

[23] Chen J, Hu RZ, Zhuang YX (2025). Natural language processing chatbot—based interventions for improvement of diet, physical activity, and tobacco smoking behaviors: systematic review. JMIR Mhealth Uhealth.

[24] Kim H, Lee S, Cho Y KMI: a dataset of korean motivational interviewing dialogues for psychotherapy.

[25] Almusharraf F, Rose J, Selby P (2020). Engaging unmotivated smokers to move toward quitting: design of motivational interviewing-based chatbot through iterative interactions. J Med Internet Res.

[26] Leeuwis L, He L (2022). Chatbot Research and Design.

[27] He L, Basar E, Wiers RW, Antheunis ML, Krahmer E (2022). Can chatbots help to motivate smoking cessation? A study on the effectiveness of motivational interviewing on engagement and therapeutic alliance. BMC Public Health.

[28] Karve Z, Calpey J, Machado C, Knecht M, Mejia MC (2025). New doc on the block: scoping review of AI systems delivering motivational interviewing for health behavior change. J Med Internet Res.

[29] Li Y, Lee KC, Bressington D (2024). A theory and evidence-based artificial intelligence-driven motivational digital assistant to decrease vaccine hesitancy: intervention development and validation. Vaccines (Basel).

[30] Moyers TB, Rowell LN, Manuel JK, Ernst D, Houck JM (2016). The Motivational Interviewing Treatment Integrity code (MITI 4): rationale, preliminary reliability and validity. J Subst Abuse Treat.

[31] Zhang C, Li R, Tan M CPsyCoun: a report-based multi-turn dialogue reconstruction and evaluation framework for Chinese psychological counseling.

[32] White J, Fu Q, Hays S, Sandborn M, Olea C A prompt pattern catalog to enhance prompt engineering with ChatGPT. https://dl.acm.org/doi/10.5555/3721041.3721046.

[33] Yang A, Xiao B, Wang B (2023). Baichuan 2: open large-scale language models. arXiv.

[34] Zeng A, Xu B, Wang B, Zhang C, Yin D (2024). ChatGLM: a family of large language models from GLM-130B to GLM-4 all tools. arXiv.

[35] Grattafiori A, Dubey A, Jauhri A (2024). The Llama 3 herd of models. arXiv.

[36] shenzhi-wang/Llama3-8B-Chinese-Chat. Hugging Face.

[37] Cui Y, Yang Z, Yao X (2023). Efficient and effective text encoding for chinese LLaMA and Alpaca. arXiv.

[38] Zheng Y, Zhang R, Zhang J, YeYanhan Y, Luo Z LlamaFactory: unified efficient fine-tuning of 100+ language models.

[39] Wu Z, Balloccu S, Kumar V Anno-MI: a dataset of expert-annotated counselling dialogues.

[40] CHART Collaborative (2025). Reporting guideline for chatbot health advice studies: the Chatbot Assessment Reporting Tool (CHART) statement. BMJ Med.

[41] Ministry of Education of the People’s Republic of China, Ministry of Science and Technology of the People’s Republic of China, National Administration of Traditional Chinese Medicine (2023). Measures for ethical review of life science and medical research involving humans [Report in Chinese]. https://www.nhc.gov.cn/wjw/c100375/202302/902b4a1dc3af4aba862a6387e6e376dc.shtml.

[42] chatopera/efaqa-corpus-zh. GitHub.

[43] ckqqqq/psy-insight: psy-insight dataset and project repository. GitHub.

[44] Xie H, Chen Y, Xing X, Lin J, Xu X PsyDT: using LLMs to construct the digital twin of psychological counselor with personalized counseling style for psychological counseling.

[45] Qiu H, He H, Zhang S, Li A, Lan Z SMILE: single-turn to multi-turn inclusive language expansion via chatgpt for mental health support.

[46] Kumar V, Rajwat PS, Medda G, Ntoutsi E, Recupero DR Unlocking LLMs: addressing scarce data and bias challenges in mental health and therapeutic counselling. https://aclanthology.org/2024.nlpaics-1.26/.

[47] Yosef S, Zisquit M, Cohen B, Klomek AB, Bar K, Friedman D (2025). The impact of fine-tuning LLMs on the quality of automated therapy assessed by digital patients. Npj Ment Health Res.

[48] Teferra BG, Huang S, Johny N (2026). Alignment of large language model responses with human therapists in motivational interviewing. JAMA Netw Open.

[49] Mahmood Z, Ali S, Zhu J A fully generative motivational interviewing counsellor chatbot for moving smokers towards the decision to quit.

[50] Christiano PF, Leike J, Brown TB, Martic M, Legg S, Amodei D Deep reinforcement learning from human preferences. https://dl.acm.org/doi/10.5555/3294996.3295184.

[51] Ouyang L, Wu J, Jiang X, Almeida D, Wainwright CL Training language models to follow instructions with human feedback. https://proceedings.neurips.cc/paper_files/paper/2022/hash/b1efde53be364a73914f58805a001731-Abstract-Conference.html.

[52] CbyerDragon/MI-LLM. GitHub.

