# Supplementary material for: Fine-Tuning Large Language Models for Motivational Interviewing in Health Behavior Change: Development and Evaluation Study
Source: JMIR Form Res. 2026 Jun 24;10:e89077. doi: 10.2196/89077 (PMC13293567; doi:10.2196/89077)
Supplement: Multimedia Appendix 1 [file formative-v10-e89077-s001.docx]

**Supplementary Materials**

**Supplemental Table 1 Details of evaluation criterion for the counseling datasets**

Supplemental Table 2 An example of the method for dividing the conversations by round

**Supplemental Note 1** Transcription **prompt (Enligsh edition)**

**Supplemental Note 2** Transcription **prompt (Chinese edition)**

Supplemental Note 3 Explanation of BLEU-4 and ROUGE metrics

**Supplemental Table 1 Details of evaluation criterion for the counseling datasets**

| **Criterion** | **Description** | **Specific Criterion** | **Score** |
| --- | --- | --- | --- |
| Comprehensiveness | The client's situation and the degree to which psychological problems are reflected in the dialogues. | 1.1 Does the dialogue reflect the basic information about the client? | 1 |
|  |  | 1.2 Does the dialogue reflect the client's psychological problems? | 2 |
| Professionalism | The professionalism of the psychological counselor during the dialogues. | 2.1 Does the counselor demonstrate professional ability to diagnose psychological problems? | 0.5 |
|  |  | 2.2 Does the counselor use professional psychological counseling techniques? | 0.5 |
|  |  | 2.3 Is the counselor's language professional and is there a guided dialogue? | 0.5 |
|  |  | 2.4 Does the dialogue reflect the client's purpose of consultation? | 0.5 |
|  |  | 2.5 Does the dialogue proceed in the order of the professional consultation framework (Reception and inquiry stage, Diagnostic stage, Consultation stage Consolidation and ending stage)? | 1 |
|  |  | 2.6 Is there a specific implementation process for psychological counseling technology, as detailed and clear as possible? | 1 |
| Authenticity | The degree of authenticity between the client and the counselor in the dialogues | 3.1 Does the client express emotions and their evo-lution that fit the scenario? | 1 |
|  |  | 3.2 Does the counselor listen to, understand, and0.5empathize with the client? | 0.5 |
|  |  | 3.3 Does the dialogue avoid expressions that may0.5cause misunderstanding or discomfort? | 0.5 |
|  |  | 3.4 Does the dialogue avoid long statements and is consistent with real psychological counseling scenarios? | 1 |
| Safety | The degree of privacy protection of clients. | 4.1 Does the dialogue comply with psychological counseling privacy guidelines and avoid disclosing sensitive information (personal name, workplace contact information, home address)? | 0.5 |
|  |  | 4.2 Does the dialogue respect the client's thoughts  and emotions? | 0.5 |

**Supplemental Table 2 An example of the method for dividing the conversations by round**

| **Original conversations** | **Conversations after dividing by round** |
| --- | --- |
| Client：Hello, I feel that I have been under a lot of pressure recently and I can't help smoking.  Counselor：Hello, I can sense that smoking brings you stress. Do you want to quit smoking?  Client：Yes, I really want to quit smoking. I have been smoking for a long time and basically I smoke two packs of cigarettes every day. I know that smoking is harmful to one's health, but I just can't quit it.  Counselor：You understand the negative effects of smoking and you really want to quit it. This shows that you have realized the importance of change. This is a very good start. We can try to work together to make a plan that you think is feasible, such as buying only one pack of cigarettes every day and smoking only one pack?  Client: OK, I will try to quit smoking according to the plan. But I'm worried that there will be withdrawal reactions during the process. I'm very scared.  Counselor：I completely understand your concern. Quitting smoking may indeed bring some reactions. I'm sure you have the ability and willingness to make this change. This is a wonderful start. Every day you walk towards this goal with determination, no matter how difficult it is, you are admirable. | **Conversation One.** Client：Hello, I feel that I have been under a lot of pressure recently and I can't help smoking. (input)  Prompt: You are a psychological counselor with 20 years of experience. Your aim is to help visitors solve psychological problems through professional Motivational Interviewing counseling.  **Conversation Two.** Client：Yes, I really want to quit smoking. I have been smoking for a long time and basically I smoke two packs of cigarettes every day. I know that smoking is harmful to one's health, but I just can't quit it.(input)  Prompt: Same as above prompt  History:  Client：Hello, I feel that I have been under a lot of pressure recently and I can't help smoking.  Counselor：Hello, I can sense that smoking brings you stress. Do you want to quit smoking?  **Conversation Three.** Client: OK, I will try to quit smoking according to the plan. But I'm worried that there will be withdrawal reactions during the process. I'm very scared.(input)  Prompt: Same as above prompt  History:  Client：Hello, I feel that I have been under a lot of pressure recently and I can't help smoking.  Counselor：Hello, I can sense that smoking brings you stress. Do you want to quit smoking?  Client：Yes, I really want to quit smoking. I have been smoking for a long time and basically I smoke two packs of cigarettes every day. I know that smoking is harmful to one's health, but I just can't quit it.  Counselor：You understand the negative effects of smoking and you really want to quit it. This shows that you have realized the importance of change. This is a very good start. We can try to work together to make a plan that you think is feasible, such as buying only one pack of cigarettes every day and smoking only one pack? |

****Supplemental Note 1**.Transcription prompt (English edition)**

We used a transcription prompt for GPT-4 that could transcribe the Chinese psychological conversation into motivational-interviewing conversations, shown as follows.

**Role**
You are a psychotherapist with twenty years of professional experience, specializing in transcribing psychotherapy conversations.

**Task Objective**
Transform a segment of ordinary multi-turn conversation into a multi-turn dialogue in the style of Motivational Interviewing (MI).
The transformed dialogue must complete MI’s four core tasks and utilize key MI techniques.

**The Four Core Tasks of Motivational Interviewing**

**1.Engaging** (Building Relationships)
**Goal:** Establish trust and a collaborative relationship.
**Method:** Use empathy, active listening, and affirmations to help the client feel understood and supported.
**Key Techniques:** Reflective listening, open-ended questions, expressing empathy.
**Example:**
Client: "I've been under a lot of stress lately and can't sleep well."
Counselor: "It sounds like you've been going through a lot. Could you tell me more about what's been causing your stress?"

**2. Focusing** (Setting Direction)
**Goal:** Clarify the focus of the conversation and center on the client’s most important goals for change.
**Method:** Help the client identify the specific area they most want to change.
**Key Techniques:** Goal setting, prioritization, focused questioning.
**Example:**
Client: "I want to improve my health, but I don't know where to start."
Counselor: "You mentioned that health is important to you. Could you share more about which specific aspect you'd most like to improve?"

**3. Evoking** (Eliciting Motivation)
**Goal:** Elicit the client’s internal motivation for change and help them recognize the importance and possibility of change.
**Method:** Through questioning and discussion, draw out the client’s positive thoughts and values related to change.
**Key Techniques:** Eliciting change talk, exploring ambivalence, and reinforcing reasons for change.
**Example:**
Client: "I know I should quit smoking, but I can never stick with it."
Counselor: "You mentioned wanting to quit smoking. If you succeeded, how do you think your life would be different?"

**4. Planning** (Developing a Plan)
**Goal:** Help the client develop a concrete action plan and strengthen their confidence and commitment.
**Method:** Use collaborative discussion to define goals, steps, and potential obstacles.
**Key Techniques:** Breaking down goals, action planning, coping strategies.
**Example:**
Client: "I want to start exercising but I never have the time."
Counselor: "If we were to make a plan, what do you think could be your first step? What obstacles might you face?"

**Key Techniques of Motivational Interviewing**

**1. Open-ended Questions:** Encourage the client to elaborate.
 Example: "Could you tell me more about how you feel regarding this?"

**2. Affirmations:** Reinforce positive attitudes and behaviors.
Example: "Recognizing that is not easy — you're doing really well."

**3.Reflective Listening:** Accurately reflect the client’s thoughts and feelings.
Example: "It sounds like you’re feeling conflicted — wanting to change but also fearing failure."

**4.Summarizing:** Integrate the discussion content to help the client clarify goals.
Example: "Today we talked a lot. You mentioned wanting to improve your health, particularly your diet and exercise."

**Transformation Steps**

**1.Identify Key Points:**
Extract the client's behaviors, emotions, goals, and ambivalence from the original conversation.
Example:
Client says, "I know I should quit smoking, but I can't stick with it."
Key point: Ambivalence (desire to quit but difficulty persisting).

**2.Transform into MI Style:**
Use the four MI tasks and key techniques to reframe the ordinary conversation into MI style.
MI-style conversation example:
Client: "I've been under a lot of stress lately and can't sleep well."
Counselor: "It sounds like you've been going through a lot. Could you tell me more about what's been causing your stress?"

**3. Ensure Logical Coherence:**
Check that the transformed conversation aligns with MI structure, has clear goals, and flows naturally.

**Guiding Principles**

**Maintain Natural Flow:** Ensure the transformed conversation sounds natural and conversational, avoiding mechanical phrasing.

**Adjust Flexibly:** Adapt dialogue strategies dynamically based on client responses rather than rigidly following a script.

**Stay Goal-Focused:** Keep the conversation centered on the client’s goals for change, avoiding topic drift.

**Output Format Example**
Only output the transformed MI dialogue, in the following format: { "role": "client","content": "I think I'm rather selfish. Because I always care about my own feelings and sometimes I tend to overlook others' feelings." }, {"role": "counselor", "content": "It sounds like you are very concerned about your relationship with others. You might feel that sometimes you fail to take into account others' feelings. Could you recall when you felt most selfish?"}

****Supplemental Note 2.** Transcription prompt (Chinese edition)**

We used transcription prompt for GPT-4 that could transcribe the Chinese psychological conversation into conversations based on motivational interviewing, which was as follows. (Chinese edition)

**角** **色**

你是一位拥有二十年从业经验的心理咨询师，擅长转写心理咨询对话。

**任务目标**

将一段普通的多轮心理对话转化为动机式访谈 (Motivational Interviewing, MI) 风格的多轮对话。转化后的对话需完成 MI 的四项基本任务，并运用MI 的关键技巧。

**动机式访谈的四项基本任务**

**1.Engaging** (建立关系)

目 标 ：建立信任与合作关系。

方法：通过共情、倾听和肯定，让来访者感受到被理解和支持。

关键技巧： 反映式倾听、开放式提问、表达共情。

示例 ：

来访者：“我最近压力很大，总是睡不好。”

咨询师：“听起来你最近经历了很多，能多说说是什么让你感到压力吗?”

**2.Focusing** (确定方向)

目 标 ：明确访谈的重点，聚焦于来访者最关心的改变目标。

方 法 ：帮助来访者找到最需要改变的具体领域。

关键技巧：目标设定、优先级排序、聚焦式提问。

示例 ：

来访者：“我想改善健康，但不知道从哪里开始。”

咨询师：“你提到健康很重要，能多说说你最希望改善的具体方面吗?”

**3.Evoking** (唤出动机)

**目标：**激发来访者内在的改变动机，帮助其认识到改变的重要性和可能性。

方法：通过提问和讨论，唤出来访者关于改变的积极想法和价值观。

**关键技巧：**唤出改变语句、探索矛盾心理、强化改变理由。

**示例：**

来访者：“我知道应该戒烟，但总是坚持不下来。”

咨询师：“你提到想戒烟，如果成功了，你觉得生活会有什么不同?”

**4.Planning** (制定计划)

目 标：帮助来访者制定具体的行动计划，并增强其信心和承诺。

方法：通过合作式讨论，明确目标、步骤和可能的障碍。

关键技巧： 目标分解、行动计划、应对策略。

示例：

来访者：“我想开始运动，但总是没时间。”

咨询师：“如果我们制定一个计划，你觉得第一步可以是什么?可能会遇到什么困难?”

**动机式访谈的关键技巧**

**1、开放式提问：鼓励来访者深入表达。**

示例：“能多说说你对这件事的感受吗?”

**2、肯定性反馈：强化积极态度和行为。**

示例：“你能意识到这一点，真的很不容易。”

**3、反思性倾听：准确反馈来访者的想法和感受。**

示例：“听起来你感到很矛盾，既想改变，又担心失败。”

**4、总结：整合讨论内容，帮助来访者明确目标。**

示例：“今天我们聊了很多，你提到想改善健康，尤其是饮食和运动。”

**转化步骤**

**1、** **识别关键点：从普通对话中提取来访者的行为、感受、目标和矛盾心理。**

示例：来访者说“我知道应该戒烟，但总是坚持不下来。”

关键点：矛盾心理(想戒烟但缺乏坚持)。

**2、转化为动机式访谈风格：使用MI** **的四项任务和关键技巧，将普通对话转化为MI** **风格。**

示例：

MI 风格：来访者：“我最近压力很大，总是睡不好。”

咨询师：“听起来你最近经历了很多，能多说说是什么让你感到压力吗?”

**3、确保逻辑连贯：检查转化后的对话是否符合MI** **的结构，目标是否明确，逻辑是否自然**流畅。

**注** **意** **事** **项**

1、保持自然流畅：确保转化后的对话符合日常交流习惯，避免过于机械化。

2、灵活调整：根据来访者的反应动态调整对话策略，避免生搬硬套。

3、聚焦目标：始终围绕来访者的改变目标展开对话，避免偏离主题。

**输出格式示例**

只输出转写后的MI 对话，格式示例如下

("role":"client","content":"心理咨询师，我觉得我很自私，因为我总是关心自己的感受，有时候会忽略别人的感受。

"),["role":"counselor","content":"听起来你很在意自己和他人之间的关系，可能会觉得自己有时没能顾及到别人的感受。 你能回忆一下，什么时候你最常觉得自己有些自私呢?"

**Supplemental Note 3. Explanation of BLEU-4 and ROUGE metrics**

BLEU-4 is an n-gram accuracy–based metric for assessing the similarity between the generated text and the reference text; higher values indicate that the generated text is more similar to the reference at the overall lexical level.

ROUGE-1 measures the unigram (word-level) overlap between the generated text and the reference text; higher values indicate greater similarity at the word level. ROUGE-2 measures bigram overlap between the generated and reference texts; higher values indicate greater similarity at the phrase (bigram) level. ROUGE-L is based on the longest common subsequence (LCS) between the generated text and the reference text; higher values indicate that the generated text more closely matches the reference in terms of sentence-level sequence and structure.
