# Supplementary material for: Fine-Tuning Large Language Models for Motivational Interviewing in Health Behavior Change: Development and Evaluation Study
Source: JMIR Form Res. 2026 Jun 24;10:e89077. doi: 10.2196/89077 (PMC13293567; doi:10.2196/89077)
Supplement: Checklist 1 [file formative-v10-e89077-s002.docx]

**Appendix 2. Fillable CHART Checklist**

| **HEADING** | **#** | **CHART CHECKLIST ITEM** | **Page #*** |
| --- | --- | --- | --- |
| **Title & Abstract** |  |  |  |
| **Title** | **1a** | State that the study is assessing one or more generative AI-driven chatbots for clinical evidence or health advice. | P.1 |
| **Abstract/Summary** | **1b** | Apply a structured format, if applicable. | P.1 |
| **Introduction** |  |  |  |
| **Background** | **2a** | State the scientific background, rationale, and healthcare context for evaluating the generative AI-driven chatbot(s), referencing relevant literature when applicable. | P.2 |
|  | **2b** | State the aims and research questions including the target audience, intervention, comparator(s), and outcome(s). | P.2 |
| **Methods** |  |  |  |
| **Model Identifiers** | **3a** | State the name and version identifier(s) of the generative AI model(s) and chatbot(s) under evaluation, as well as their date of release or last update. | P.3 |
|  | **3b** | State whether the generative AI model(s) and chatbot(s) are open-source or closed-source/proprietary. | P.3 |
| **Model Details** | **4a** | State whether the generative AI model was a base model or a novel base model, tuned model, or fine-tuned model. | P.3-4 |
|  | **4b** | If a base model is used, cite its development in sufficient detail to identify the model. | P.3 |
|  | **4c** | If a novel base model, tuned model, or fine-tuned model is used, describe the pre- and/or post-implementation/deployment data and parameters. | P.4, P.6 |
| **Prompt Engineering** | **5a** | Describe the evolution of study prompt development. | P.3 |
|  | **5ai** | Describe the sources of prompts. | P.3 |
|  | **5aii** | State the number and characteristics of the individual(s) involved in prompt engineering. | P.3 |
|  | **5aiii** | Provide details of any patient and public involvement during prompt engineering. | N/A; no patients or public involved |
|  | **5b** | Provide study prompts. | Multimedia Appendix 1 |
| **Query Strategy** | **6a** | State route of access to generative AI model. | P.3-4 |
|  | **6b** | State the date(s) and location(s) of queries for the generative AI-driven chatbot(s) including the day, month, and year as well as city and country. | P.3-4 |
|  | **6c** | Describe whether prompts were input into separate chat session(s). | P.4 |
|  | **6d** | Provide all generative AI-driven chatbot output/responses | P.4, P.11 |
| **Performance Evaluation** | **7a** | Define the ground truth or reference standard used to define successful generative AI-driven chatbot performance. | P.4-5 |
|  | **7b** | Describe the process undertaken for generative AI-driven chatbot performance evaluation. | P.4-5 |
|  | **7bi** | State the number and characteristics of team members involved in performance evaluation. | P.5 |
|  | **7bii** | Provide details of any patients and public involvement during the evaluation process. | N/A; no patients or public involved in evaluation |
|  | **7biii** | State whether evaluators were blinded to the identity of the generative AI-driven chatbot(s) under assessment. | P.5 |
| **Sample Size** | **8** | Report how the sample size was determined. | P.4-5 |
| **Data Analysis** | **9a** | Describe statistical analysis methods, including any evaluation of reproducibility of generative AI-driven chatbot responses. | P.4-5 |
|  | **9ai** | Report the measures used for performance evaluation. | P.4-5 |
| **Results** |  |  | P.6-9 |
|  | **10a** | Report the alignment between generative AI-driven chatbot output and ground truth or reference standard using quantitative or mixed methods approaches as applicable. | P.7-9 |
|  | **10b** | For responses deviating from the ground truth or reference standard, state the nature of the difference(s). | P.9-10 |
|  | **10c** | Report the assessment for potentially harmful, biased, or misleading responses. | P.3, P.10 |
| **Discussion** |  |  | P.9-10 |
|  | **11a** | Interpret study findings in the context of relevant evidence. | P.9-10 |
|  | **11b** | Describe the strengths and limitations of the study. | P.10 |
|  | **11c** | Describe the potential implications for practice, education, policy, regulation, and research. | P.10 |
| **Open Science** |  |  |  |
| **Disclosures** | **12a** | Report any relevant conflicts of interest for all authors. | P.11 |
| **Funding** | **12b** | Report sources of funding and their role in the conduct and reporting of the study. | P.11 |
| **Ethics** | **12c** | Describe the process undertaken for ethical approval. | P.5-6 |
|  | **12ci** | Describe the measures taken to safeguard data privacy of patient health information, as applicable. | P.5-6 |
|  | **12cii** | State whether permission/licensing was obtained for the use of original, copyrighted data. | P.5-6 |
| **Protocol** | **12d** | Provide a study protocol. | N/A |
| **Data availability** | **12e** | State where study data, code repository, and model parameters can be accessed. | P.11 |

*If in supplementary appendix, indicate “supp” and appendix #, if applicable.
